# Decoding interactions between biofilms and DNA nanoparticles

**DOI:** 10.1016/j.bioflm.2025.100260

**Published:** 2025-02-06

**Authors:** Alexandra Sousa, Rutuparna Kulkarni, Mona Johannessen, Thorsten Wohland, Nataša Škalko-Basnet, Sybil Obuobi

**Affiliations:** a Drug Transport and Delivery Research Group, Department of Pharmacy, UIT The Arctic University of Norway, Tromsø, Norway; b Department of Biological Sciences, National University of Singapore, Singapore; c Centre for BioImaging Sciences, National University of Singapore, Singapore; d Host Microbe Interaction Research Group, Department of Medical Biology, UIT The Arctic University of Norway, Tromsø, Norway

**Keywords:** *P. aeruginosa biofilms*, DNA nanoparticles, Nucleic acid, Chitosan, Microfluidics, Diffusion coefficient

## Abstract

Biofilms present a great challenge in antimicrobial therapy due to their inherent tolerance to conventional antibiotics, promoting the need for advanced drug delivery strategies that improve therapy. While various nanoparticles (NPs) have been reported for this purpose, DNA-based NPs remain a largely unexploited resource against biofilm-associated infections. To fill this gap and to lay the groundwork for their potential therapeutic exploitation, we investigated the diffusion, penetration, and retention behaviors of three DNA-based nanocarriers —plain or modified—within *P. aeruginosa* biofilms. Watson-Crick base pairing or hydrophobic interactions mediated the formation of the plain NPs whilst electrostatic interaction enabled optimization of coated NPs via microfluidic mixing. We assessed the interactions of the nanocarriers with biofilm structures via Single Plane Illumination Microscopy – Fluorescence Correlation Spectroscopy (SPIM-FCS) and Confocal Laser Scanning Microscopy (CLSM). We demonstrate the impact of microfluidic parameters on the physicochemical properties of the modified DNA NPs and their subsequent distinct behaviors in the biofilm. Our results show that single stranded DNA micelles (ssDNA micelle) and tetrahedral DNA nanostructures (TDN) had similar diffusion and penetration profiles, whereas chitosan-coated TDN (TDN-Chit) showed reduced diffusion and increased biofilm retention. This is attributable to the relatively larger size and positive surface charge of the TDN-Chit NPs. The study shows first and foremost that DNA can be used as building block in drug delivery for antibiofilm therapeutics. Moreover, the overall behavioral findings are pivotal for the strategic selection of therapeutic agents to be encapsulated within these structures, possibly affecting the treatment efficacy. This research not only highlights the underexplored potential of DNA-based NPs in antibiofilm therapy but also advocates for further studies using different optimization strategies to refine these nanocarrier systems for targeted treatments in biofilm-related infections.

## Graphical abstract

**Figure:**
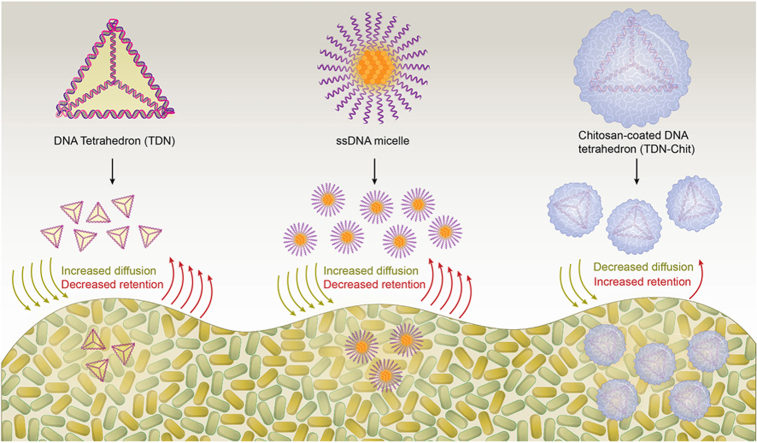

## 1 Introduction

The persistent threat of bacterial infections has long imposed a significant burden on global health systems, further aggravated by bacterial ability to survive antimicrobial therapy. Notably, antimicrobial resistance was linked to approximately 5 million deaths in 2019, with projections suggesting this number could rise to 10 million by 2050, underscoring the urgent need for effective healthcare interventions [1,2]. Among the most concerning are the ESKAPE pathogens: *Pseudomonas aeruginosa*, *Enterococcus faecium*, *Staphylococcus aureus*, *Klebsiella pneumoniae*, *Acinetobacter baumannii*, and *Enterobacter* spp., which are deemed worthy of increased attention [3]. These and many other pathogens possess an ability to form biofilms—a complex aggregation of bacteria encased within a robust extracellular polymeric substance (EPS) composed of polysaccharides, proteins, lipids, and extracellular DNA [4]. The biofilm formation poses a formidable challenge in treatment due to the enhanced antibiotic tolerance conferred by (but not limited to) the biofilm matrix. For example, studies have shown that in *P. aeruginosa*, the biofilm matrix can sequester antibiotics like tobramycin at its periphery, preventing their penetration into deeper layers [5]. The unique physicochemical properties of various antibiotic classes further complicate their diffusion and retention within biofilms. Aminoglycosides such as gentamicin, amikacin, and tobramycin, are large molecules that are electrostatically attracted to matrix components, whereas fluoroquinolones and macrolides are characterized by low retention, diminishing their effectiveness [6,7]. Additionally, the slow growth rate of bacteria within biofilms renders antibiotics that require active bacterial replication less effective [8]. These factors significantly contribute to the health risks associated with bacterial biofilms, highlighting the need for innovative drug delivery strategies.

One promising approach to overcoming these challenges is the use of nanoparticles (NPs) for drug delivery. Research has extensively explored different materials for the fabrication of these structures, including lipids, polymers, and inorganic substances like gold and silver, as well as hybrid structures combining these materials [[9], [10], [11], [12]]. These NPs can be engineered for targeted delivery, to respond to specific stimuli, or to enhance the payload amount inside biofilms [[13], [14], [15]]. However, challenges such as limited translational potential and toxicity remain [16,17]. As an alternative, oligonucleotides like deoxyribonucleic acid (DNA) have garnered interest due to their ease of fabrication, programmability, and biocompatibility, presenting a viable option as drug delivery systems [[18], [19], [20]]. The ability to design DNA sequences that can hybridize in a controlled manner and be functionalized with additional capabilities further enhances their potential [[21], [22], [23]]. Moreover, it has been extensively studied in fields such as oncology [[24], [25], [26], [27]] and diagnostics [28,29].

Although DNA nanotechnology has been rigorously investigated in various fields, its application for addressing bacterial infections, particularly within biofilms, has not been thoroughly explored. The potential of nanotechnology as a therapeutic strategy for biofilms is recognized, yet the specific interactions between NPs and biofilms remain insufficiently understood, likely due to the inherent heterogeneity of biofilms [15]. Previous studies, such as the one conducted by Deiss-Yehiely et al., have begun to shed light on how different NPs properties affect their interactions with key components of *P. aeruginosa* biofilms [30]. Other studies have advanced our understanding of biofilm-NP dynamics [[31], [32], [33], [34], [35]], however, a significant gap in research remains, especially regarding the use of DNA-based nanostructures. Our previous work has demonstrated the biofilm penetration capabilities of polymyxin-B loaded DNA micelles [36], yet this promising area remains largely under-researched. This study seeks to fill this gap by exploring the interaction, diffusion and retention of a range of drug-free DNA NPs distinct in their physicochemical properties with or within *P. aeruginosa* biofilms. To the best of our knowledge, this is the first study to evaluate how various DNA-based NPs interact with bacterial biofilms, potentially setting the stage for their future application for biofilm therapy.

## 2 Results and discussion

**Fig. 1:**
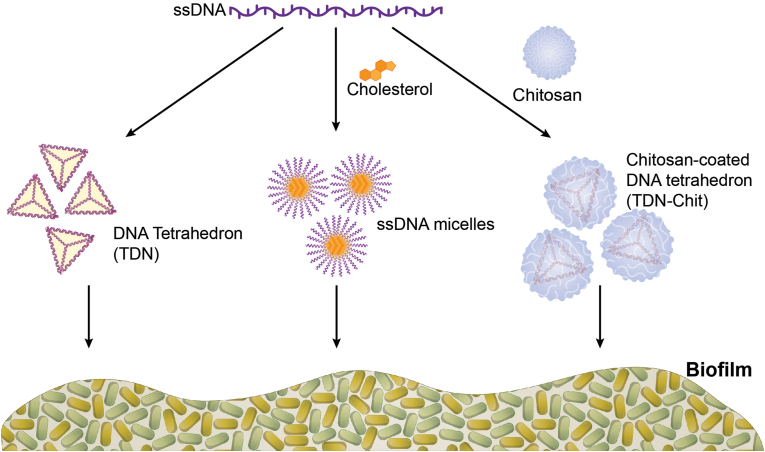
**Schematic representation of DNA-based NPs.** The formation of a DNA tetrahedron (TDN) results from the rational design of four oligonucleotide sequences of single stranded DNA (ssDNA) that hybridize together in the shape of a tetrahedron (left). The formation of ssDNA micelles results from the addition of cholesterol-modified ssDNA strands that spontaneously self-assemble into a micelle at concentrations above critical micelle concentration (center). Chitosan-coated DNA tetrahedron (TDN-Chit) results from the mixing of a chitosan solution with pre-formed TDN through manual addition or using a microfluidics device (right).

**Fig. 2:**
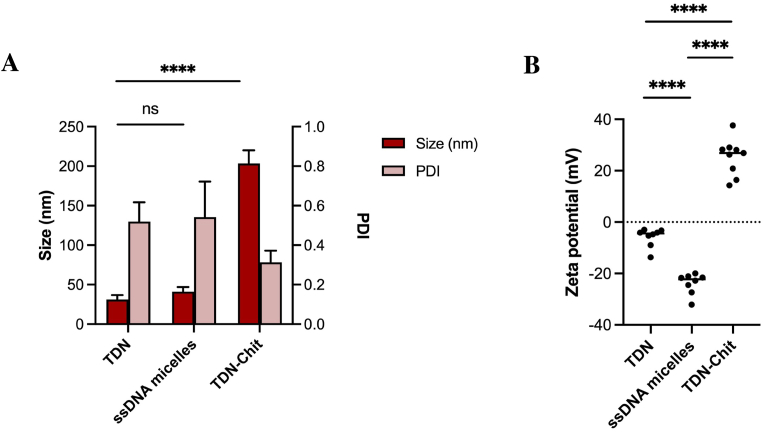
**Characterization of different NPs using DLS**. **A)** Size and PDI of tetrahedral DNA (TDN), cholesterol-modified ssDNA micelles (ssDNA micelles) and chitosan-coated TDN (TDN-Chit); **B)** Zeta potential of TDN, ssDNA micelles and TDN-Chit.

To generate a diverse library of DNA-based nanostructures with varying physicochemical characteristics, we initially focused on the production and optimization of both unmodified and modified DNA NPs using different preparation techniques (Fig. 1). We report on three DNA NPs namely single stranded DNA micelles (ssDNA micelles), DNA tetrahedral nanoparticles (TDN) and chitosan-coated DNA tetrahedral nanoparticles (TDN-Chit). The resulting nanostructures varied in size (Fig. 2A) and surface charge (anionic and cationic) (Fig. 2B); some structures comprised additional functional groups (i.e., cholesterol) or polymers (i.e., chitosan).

Fabrication of the DNA tetrahedrons (TDN) was achieved via thermal annealing as previously described [[37], [38], [39]]. As the pristine DNA NP, the pyramidal structured TDN NPs were prepared without any modifications on the oligonucleotide. The hydrodynamic size of the TDN was determined to be 31 ± 5 nm, with a polydispersity index (PDI) of 0.52 ± 0.10 and a negative surface charge of −5.9 ± 3.7 mV, aligning with values reported in similar studies [37,40].

Subsequently, we aimed to enhance the hydrophobicity of the nanostructures, as it has been shown that hydrophobic NPs exhibit improved antibiofilm activity [41]. To this end, we prepared DNA micelles (ssDNA micelles) through the spontaneous self-assembly of cholesterol-modified single-stranded DNA [42,43]. The critical micelle concentration (CMC) was determined by monitoring absorbance changes within a range of increasing cholesterol-modified ssDNA concentrations. Other studies have shown that the absorbance is linear until the CMC point is reached, and for concentrations higher than the CMC, an inflection point is observed as the micellization process causes a shift in linearity [44,45]. Our results show a linearity change after 11.5 μM (Fig. S1), consistent with previous studies that identified a similar critical aggregation point at 10 μM [43], and corroborated by reports that no micelles formed below 1 μM [42]. For further experiments, we used a concentration of 20 μM to ensure micelle formation well above the CMC. At this concentration, the ssDNA micelles exhibited a size of 41 ± 6 nm, a PDI of 0.54 ± 0.18, and a significantly more negative surface charge of −23.9 ± 4.0 mV compared to the TDN. This variation is likely attributed to the difference in oligonucleotide concentrations used to prepare the TDN (1 μM) and ssDNA micelles (20 μM).

To explore the impact of surface chemistry on the behavior of DNA-based NPs, the TDN NPs were coated with chitosan. Chitosan is a cationic polymer known for its high number of amine groups which can be protonated at acidic pH, exhibiting ability to form complexes with negatively charged oligonucleotides has been well-documented in gene therapy [46]. Typically, oligonucleotide-chitosan complexes are prepared using bulk mixing methods, where solutions of DNA and chitosan are combined, leading to the spontaneous formation of complexes through electrostatic interactions.

Adopting this approach, we created DNA-Chitosan NPs (TDN-Chit) by mixing pre-formed TDNs with a chitosan solution (pH = 6.03 ± 0.69). Subsequent zeta potential measurements confirmed a successful reversal of charge from negative to positive (+25.3 ± 7.1 mV). The size of these NPs was 203 ± 17 nm, with a low polydispersity index (PDI) of 0.31 ± 0.06, aligning with sizes reported in the literature for similar complexes (150–300 nm) [47]. Notably, the chitosan coating resulted in NPs significantly larger than the uncoated TDN and ssDNA micelles, posing a challenge in isolating the effects of charge modification on biofilm interactions. However, larger particles have been shown to be effective in drug delivery applications for bacterial biofilms [[48], [49], [50]]. Therefore, we considered the TDN-Chit structure suitable for further exploration of DNA-based NPs interactions with biofilms.

**Fig. 3:**
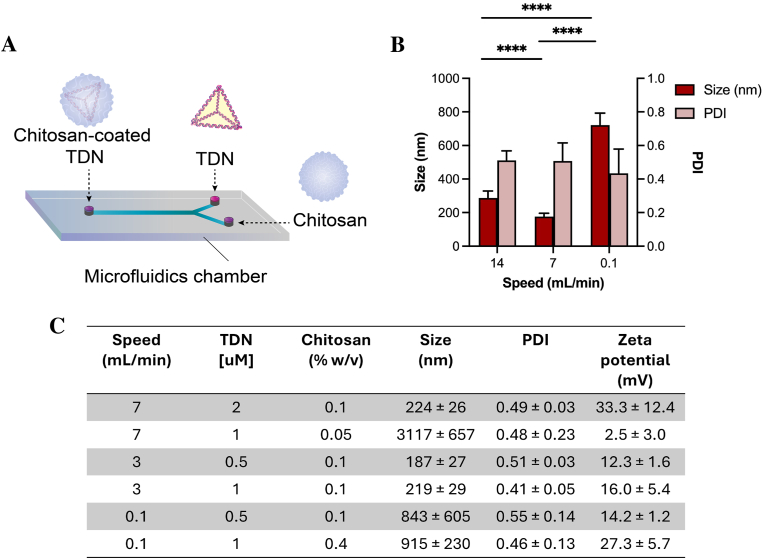
**Characterization of TDN-Chit fabricated via microfluidics**. **A)** Schematic representation of the microfluidics device used to coat TDN with chitosan to generate TDN-Chit. The TDN suspension and chitosan solutions were added to the microfluidics device through syringe injection at speeds controlled with the use of a pump **B)** Effect in size and PDI of TDN-Chit manufactured using different speeds of injection and constant concentration of TDN and chitosan; **C)** Effect in size, PDI and zeta potential of TDN-Chit made using varying speeds and concentrations.

To achieve more precise control over the size of the TDN-Chit complexes, we explored an alternative fabrication strategy utilizing microfluidic technology, which has been recognized for its capability to downsize and control NPs dimensions [51,52]. In this context, different microfluidic apparatus can be used [53], and here we employed a slightly modified Y-shaped microfluidic device (Fig. 3A). This setup allows for the simultaneous injection of two solutions at controlled flow rates, converging in a central channel where the mixing occurs [54]. The dynamics within this system are heavily influenced by the interfacial forces between the solutions, which are in turn governed by the flow rates of the injected solutions.

We investigated the effect of varying flow rates on the size of the TDN-Chit complexes, with the results presented in Fig. 3B. At a lower flow rate of 0.1 mL/min, we observed the formation of large aggregates with an average size of 721 ± 72 nm. Conversely, at higher flow rates of 7 and 14 mL/min, the particle sizes were significantly reduced to 177 ± 20 nm and 287 ± 42 nm, respectively. Across all tested conditions, the zeta potential remained consistently positive, ranging from +25 to +35 mV (Fig. S2). The observed size variations among the different flow rates did not follow a linear trend, indicating complex dynamics during the mixing process.

Previous studies have noted that higher flow rates generally lead to smaller NPs sizes due to reduced mixing times, which promote the formation of more compact structures [55,56]. This aligns with our observations, where increasing the flow rate from 0.1 mL/min to 3 mL/min and 7 mL/min resulted in smaller NPs. Liu et al. also reported a non-linear relationship between NPs size and flow rate, initially observing an increase in size followed by a decrease as the flow rate continued to rise [57]. It is relevant to underline that our work compared a broader flow rate range, whereas the study in question focused on a narrower scale. Nevertheless, the authors agree that higher volumetric flow rates enhance the mixing of fluids, but once this mixing strength threshold is achieved, residence time becomes relevant, and therefore the different trends could be explained by the tradeoff between the mixing efficiency and residence time. The final physicochemical characteristics of the nanoparticles are highly reliant on the flow type in the microfluidics device and associated Reynolds number, and the reader is directed to more extensive literature on the subject [58].

The choice of microfluidic device, the interaction mechanisms of the NPs components, the total and relative flow rates, as well as the dimensions and geometry of the mixing channel, including inlet confluence angles, all play significant roles in controlling NPs size [59]. Given these complexities, further investigations are necessary to fully elucidate the impact of the flow rate on the size control of chitosan-oligonucleotide complexes fabricated with Y-type microfluidic devices, in which the TDN suspension and chitosan solutions were in syringes attached to a pump, with which we were able to control the speed of addition of both components in the microfluidics chamber.

Additional factors, such as the concentration of the components involved, also significantly influence the size of oligonucleotide-chitosan complexes [60]. To explore this aspect, we evaluated the impact of varying DNA:Chitosan ratios at two different flow rates previously tested (Fig. 3C). Notably, increasing TDN concentration in a 2-fold manner resulted in sizes of 224 ± 12.4 nm, and surface charge of 33.3 ± 12.4 mV at a flow rate of 7 mL/min, however reducing the chitosan concentration by half resulted in the formation of exceptionally large aggregates (3391 ± 708 nm). The latter phenomenon can likely be attributed to the near-neutral zeta potential (+3.0 ± 3.0 mV) observed under these conditions, which diminishes repulsive forces between particles and promotes aggregation. However, these findings highlight the importance of optimizing the DNA:Chitosan ratio within the same speed. For example, lowering the speed to 3 mL/min resulted in similar formulations whilst maintaining a constant chitosan concentration, varying TDN concentration from 0.5 to 1 μM led to particles with 187 ± 27 nm and 219 ± 29 nm, with charges of 12.3 ± 1.6 and 16.0 ± 5.4 mV, respectively.

Conversely, at the lower flow rate of 0.1 mL/min, the formation of large aggregates appeared to be less sensitive to changes in the concentrations of either oligonucleotide or chitosan, as large aggregates persisted regardless of the concentration adjustments. Despite maintaining positive zeta potentials (around +25 mV), altering the oligonucleotide concentration by reducing it by half or increasing the chitosan concentration up to four times still resulted in the formation of substantial aggregates, with sizes of 843 ± 605 nm and 915 ± 230 nm, respectively.

These observations underscore that while higher flow rates in microfluidic devices facilitate the preparation of smaller NPs, the ratio of chitosan to DNA remains a critical factor influencing the size of the resulting complexes. Moreover, other parameters, such as the molecular weight of chitosan or its degree of acetylation, also impact the size of these complexes. For a more detailed discussion on these influences, readers are directed to additional literature [61].

It is important to note that even though no drug was loaded into the NPs in this study, we believe all three carriers would be suitable candidates as drug delivery systems. For instance, we have previously shown that ssDNA micelles can be loaded with different concentrations of polymyxin B and showed antibiofilm effect [36]. Other plain DNA based structures have shown the capacity to load antimicrobial peptides [62], including TDNs [63], with enhanced antimicrobial action. Moreover, chitosan has been widely researched for drug delivery systems either alone or coating other NPs [64,65], so we believe this coating is an ally for drug delivery systems.

Lastly, we would like to note that despite the fact that toxicity evaluations were not presented in this work, we have strong reason to believe that these particles would not present immediate signs of toxicity. Namely, previous work conducted in our group evaluated the cytotoxicity of ssDNA micelles at the same concentration were evaluated against macrophages and fibroblasts that showed no impact on cell viability, both plain and loaded with different concentrations of polymyxin B [36]. Those findings were in agreement with work conducted by other research groups using DNA based micelles with no signs of toxicity [21,66]. Extensive research has been conducted on TDN structures for different cell lines with no adverse response [67], and this structure has been generally recognized as safe, inclusively showing to promote the regeneration of various tissued in the body [[68], [69], [70], [71]]. A similar rational lead us to believe in the overall safety of chitosan [64,72], including the approval of chitosan-containing products with approval from the US Food and Drug Administration [73]. Moreover, chitosan has been extensively used in the fabrication of NPs both as sole component and as a coating agents with promising results with no obvious signs of toxicity [74,75]. Moreover, a study by Li et al. [76] reported the fabrication of a chitosan-containing hydrogel as scaffold for the recruitment of TDN for the treatment of injured articular cartilage with promising results, where in vivo data showed improved cartilage regeneration and no signs of cytotoxicity. The interaction between bacteria and various molecules, including antibiotics and NPs, has been extensively studied through the measurement of shifts in bacterial surface charge, indicated by changes in zeta potential [77,78]. Various factors influence the interaction between NPs and bacterial surfaces, such as particle size [79], the presence of hydrophobic components [80], positive charges [81], and specific shapes [82], all of which can influence the attraction between NPs and bacteria.

**Fig. 4:**
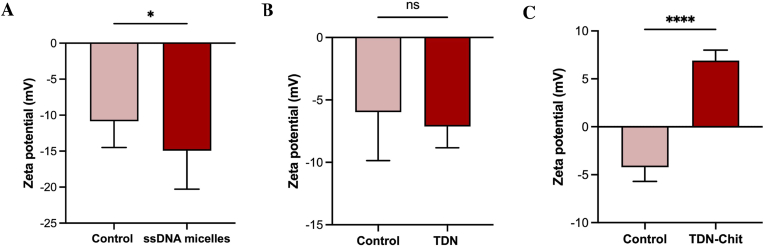
**Interaction of NPs with the surface of *P. aeruginosa***. Zeta potential values of bacterial suspensions of *P. aeruginosa* after incubation with **A)** ssDNA micelles; **B)** TDN; **C)** TDN-Chit.

Zeta potential measurements can vary based on the pH and salt concentration of the media [83,84]. In our study, we fabricated NPs in different buffers and measured the zeta potential of bacterial surfaces post-NP incubation, comparing these values to those of bacteria in the respective NP buffers, with results depicted in Fig. 4. The exposure to the TDN did not significantly alter the bacterial surface charge, with measurements showing −6.0 ± 3.9 mV and −7.1 ± 1.7 mV before and after incubation, respectively. This lack of significant change aligns with existing literature, suggesting that anionic NPs minimally interact with bacterial surfaces [81]. Previous studies have indicated that zeta potential changes can be associated with either the surface attachment or internalization of NPs [84], and prior research on TDNs suggests their potential internalization by bacteria [40,85], rather than mere surface accumulation.

Conversely, exposure to the TDN-Chit NPs, which contains positively charged chitosan, resulted in a notable increase in zeta potential from −4.2 ± 1.5 mV to +6.9 ± 1.1 mV. This outcome was anticipated, as positively charged NPs are known to interact more strongly with bacterial surfaces [81,86], and chitosan is recognized for its ability to bind to bacterial cell walls [87]. Additionally, when bacteria were exposed to micelles, there was a decrease in bacterial zeta potential from −10.8 ± 3.6 mV to −14.9 ± 5.4 mV. It is important to outline that the micelles were more anionic than the TDN, and therefore one possible explanation for greater interaction between ssDNA micelles and bacteria is the binding of NPs to the cationic sites on the bacterial surface, thus reducing the overall surface charge of bacteria, as seen in the work of Zając et al. [84]. Additionally, given the role of hydrophobic forces in NP-bacteria interactions, we hypothesize that the presence of hydrophobic components within the micelles might enhance their interaction with bacteria. This interaction could potentially counteract some of the electrostatic repulsion forces between the negatively charged bacteria and micelles. This theory is supported by previous studies indicating that anionic NPs can bind to microbial cells, possibly through hydrophobic interactions [88].

### 2.1 Nanoparticle diffusion through biofilms

Our study focused on understanding the diffusion behavior of NPs within biofilms, specifically examining their motions within the biofilm. The primary mechanisms by which particles navigate through biofilms are not entirely clear, but diffusion is widely accepted as the main driving force [32]. The diffusion process is influenced by the characteristics of both the biofilm and the NP and can be studied using Single Plane Illumination Microscopy-Fluorescence Correlation Spectroscopy (SPIM-FCS) [89].

FCS analysis provides insights into two crucial parameters that describe the mobility of fluorescent molecules: the average number of particles in the observation volume and the diffusion coefficient of these molecules. The amplitude of the autocorrelation function indicates the number of particles, while its width correlates with the diffusion coefficient. According to the Stokes-Einstein equation, the diffusion kinetics of a molecule primarily depends on its hydrodynamic radius, assuming constant viscosity and temperature. In this research, we focused on identifying characteristics of DNA-based NPs that either facilitate or hinder diffusion, using only one bacterial strain to eliminate variability due to different pathogens.

**Fig. 5:**
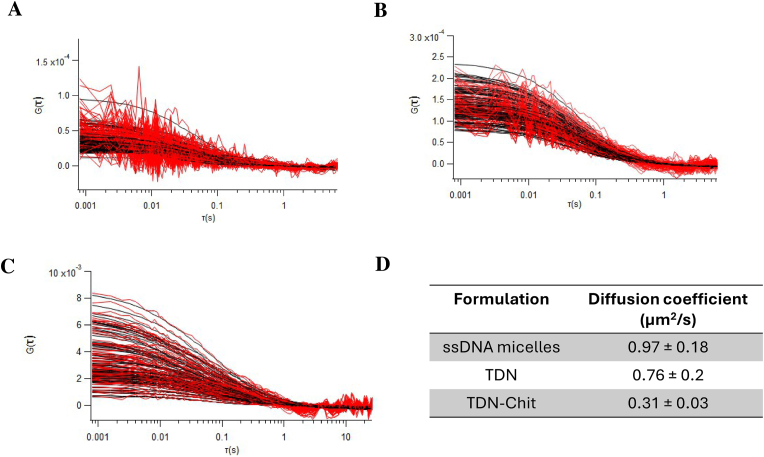
**Diffusion of NPs inside mature (72h) *P. aeruginosa* biofilms**. Autocorrelation curves of different NPs after incubation with pre-formed *P. aeruginosa* biofilms, analyzed using SPIM-FCS, namely **A)** ssDNA micelles and **B)** TDN and **C)** TDN-Chit. **D)** Average diffusion coefficient of ssDNA micelles, TDN and TDN-Chit inside *P. aeruginosa* biofilms. Results represented as mean ± standard deviation (n = 3).

We added NP solutions into preformed biofilms and measured their diffusion coefficients while analyzing their autocorrelation functions. The autocorrelation function curves for ssDNA micelles and TDN (Fig. 5A and B**,** respectively) showed a lower amplitude for ssDNA micelles compared to TDN, which can indicate a higher concentration of ssDNA micelles inside the biofilms. Even though the same concentration of fluorescent probe was used in the manufacture of each NP (as means of ensuring similar signals between the different nanostructures), the relationship between the number of particles and amplitude is complex, as it also depends on the signal-to-noise ratio. The ssDNA micelles displayed higher noise, possibly due to a lower signal-to-noise ratio or increased background noise. Thus, this analysis alone does not conclusively indicate a higher concentration of ssDNA micelles within the biofilms compared to TDN. The autocorrelation function data for TDN-Chit was quite noisy, and for this reason, for some of the measurements we performed one-particle fitting and for other a two-particle fitting was employed (Fig. S3).

Next, we calculated the diffusion coefficients for the three NPs using the 3D SPIM-FCS fitting model integrated into the ImageJ (FIJI) imaging FCS (imFCS) plugin [90]. The results, shown in Fig. 5C, indicate that ssDNA micelles and TDN have similar diffusion coefficients, at 0.97 ± 0.18 μm^2^/s and 0.76 ± 0.2 μm^2^/s, respectively. Due to the noisier autocorrelation functions of the TDN-Chit, we adopted a two-particle fitting approach to determine the presence of different particles in our sample, their fraction, and respective diffusion coefficients. We observed that a large fraction of particles (72 %) had a lower diffusion coefficient (0.34 ± 0.2 μm^2^/s), and a faster component was also present, with a D value of 20 ± 13.5 μm^2^/s. This faster component likely corresponds to fluorescently labelled DNA strands that did not form the TDN and were not encapsulated by the chitosan, and due to their smaller size, move faster. Notably, the initial one-particle fitting analysis for some datasets revealed a value of 0.31 ± 0.03 μm^2^/s, in agreement with the diffusion coefficient value for the largest fraction. This led to a statistically significant reduction in comparison to the TDN and ssDNA micelles highlights the impact of surface modifications on NP mobility within biofilms.

Considering the findings, the most notable result is the reduced diffusion coefficient observed for the TDN-Chit NPs. Two primary distinctions set TDN-Chit apart from ssDNA micelles or TDN: larger size and a positive charge. These factors are known to significantly influence the interactions between NPs and biofilms.

Previous research has underscored the importance of NP size on their ability to penetrate biofilms. Smaller NPs are generally more capable of deeper penetration. For instance, Forier et al. identified a size threshold of 100–130 nm for effective penetration in biofilms of *P. aeruginosa* and *Burkholderia multivorans* [91]. This finding suggests that larger NPs, such as those coated with chitosan, might experience reduced diffusion within biofilms.

In addition to size, the charge of NPs plays a critical role in their behavior within biofilms. Upon contact with biological environments, NPs rapidly acquire a protein corona, which significantly influences their size and the nature of their interactions with their surroundings [92]. While some studies have proposed that charge plays a minimal role in NPs diffusion [93], or that negatively charged surfaces might enhance association with biofilms [94], recent findings by Rodríguez-Suárez et al. indicate that charge effects can vary depending on the maturity of the biofilm. Specifically, they found that positively charged NPs exhibited lower diffusion coefficients in younger (48 h) *P. aeruginosa* biofilms, compared to mature (96 h) biofilms [95].

The interaction between the positively charged chitosan on TDN-Chit and the negatively charged molecules within the biofilms could also contribute to the observed reduction in diffusion. Previous studies have shown that amine-functionalized (cationic) NPs attract anionic proteins from the extracellular polymeric substances (EPS), leading to the formation of large aggregates—a phenomenon not observed with carboxylate (anionic) NPs [96]. A similar process could occur with TDN-Chit, where the amines in the chitosan attract negatively charged proteins, leading to an increase in particle size and further impeding their diffusion. This behavior is corroborated by the fitting curves of this formulation, where at longer exposure times, values for G(τ) are negative, indicating aggregation. This mechanism likely contributes to the lower diffusion coefficient values observed for TDN-Chit compared to ssDNA micelles or TDN.

It is important to consider that the NPs in this study were assembled in different buffer solutions, each containing varying ion concentrations. The biofilm matrix, characterized by its porous structure, is influenced by the degree of crosslinking among the polymers that constitute the matrix. The concentration of salts not only affects the binding of NPs to the biofilm [94] but also impacts the degree of polymer crosslinking, thereby altering pore sizes. For instance, it has been demonstrated that higher concentrations of divalent cations, such as Mg^2+^ and Ca^2+^, lead to a reduction in pore size [97]. Additionally, a known method using cation exchange resins is used to extract extracellular polymeric substances from the biofilm matrix, as the removal of cations affects the bridging of the anionic moieties in the proteins and polysaccharides in the biofilm [98]. The same logic could explain why the addition of buffers with different cation concentrations could impact the matrix, and how this difference could result in altered diffusion coefficients had the same particle been prepared in a different buffer. Given that diffusion coefficients generally decrease with increasing particle sizes, we hypothesize that this effect would be more pronounced for smaller particles (ssDNA micelles and TDN) rather than for the larger TDN-Chit. However, salt concentrations also significantly influence the final structure of NPs, potentially causing uncontrolled aggregation and changes in shape [36,42]. Therefore, there is a tradeoff between adjusting buffer strengths for optimizing NP properties and the potential impact on biofilm pore sizes. A possible method to remove buffer-induced differences in results is to use buffer exchange, however caution is advised as oligonucleotides are sensitive to ionic strength and pH [36].

The TDN was prepared in a buffer with a higher Mg^2+^ concentration, which might lead to smaller biofilm pores and, consequently, reduced diffusion compared to the ssDNA micelles. However, the differences in diffusion coefficients between ssDNA micelles and TDN were not statistically significant, suggesting that, at these concentrations, the buffer did not significantly influence the diffusion behaviors of these NPs.

Another aspect to consider is the shape of the NPs. Both ssDNA micelles and TDN possess the same surface groups, as DNA constitutes their outermost layer, and they share similar sizes and charges. The literature predominantly discusses the use of spherical NPs for antibiofilm applications, including liposomes, metallic, polymeric and solid lipid nanoparticles [[99], [100], [101], [102], [103], [104]]. However, other shapes, such as rod-shaped [105], cubes, and tetrapods [106], have also been shown to be effective against biofilms. In our study, despite the different shapes of ssDNA micelles and TDN, there was no statistically significant difference in their diffusion profiles, indicating that shape might not have a significant influence in diffusion, considering that both structures were rather small. The significant reduction in the diffusion coefficient for TDN-Chit was primarily attributed to changes in size and charge, which are well-documented factors affecting diffusion in biofilms.

### 2.2 Nanoparticle biofilm penetration and retention

**Fig. 6:**
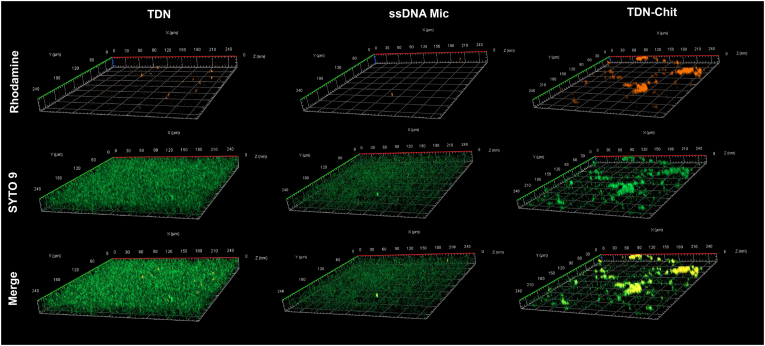
***In vitro* penetration evaluation of fluorescently labelled NPs in mature (72h) *P. aeruginosa* biofilms using z-stack images obtained with CLSM.** The biofilms were allowed to form for 72h and incubated with the different nanostructures for 2.5h. The bacteria were stained with SYTO 9 (green) and the NPs were rhodamine-labelled (red). Results show poor penetration of TDN and ssDNA micelles contrastingly to enhanced ability of TDN-Chit to penetrate mature biofilms. (For interpretation of the references to colour in this figure legend, the reader is referred to the Web version of this article.)

After determining the diffusion coefficient of each NPs, we sought to visualize their biofilm penetration and retention abilities. To achieve this, we conducted biofilm penetration assays using Confocal Laser Scanning Microscopy (CLSM). We maintained similar conditions for biofilm formation as those used for the diffusion coefficient experiments to ensure consistency in assessing general NPs behavior within the biofilm. The NPs were labelled with rhodamine, and we captured z-stack images of the SYTO9-stained biofilms after incubation with the NPs to observe their penetration, as depicted in Fig. 6.

The initial observation was that all NPs were detectable within the biofilms, albeit with varying degrees of penetration. Both ssDNA micelles and TDN showed similar extents of presence within the biofilms, correlating with their similar diffusion coefficients. The observed low red intensity for these particles suggests limited movement within the biofilm, leading us to hypothesize that not all NPs had penetrated the biofilm within the 2.5-h timeframe and the potential removal of the particles following the washing step after incubation. We noted a slight difference in biofilm density between the two, which could be attributed to the different buffers used during NPs preparation, as previously discussed in the section above. To confirm this, we incubated biofilms with the respective buffers alone for the same duration (Fig. S4), where a minor difference was observable. Given the similar small size and anionic nature of both particles, along with comparable diffusion coefficients and penetration degrees observed in confocal images, we concluded that these differences were not significant enough to exclude direct comparisons between the two. Nevertheless, to eliminate human bias in the comparison of red intensity inside the biofilms, and as means of normalizing NPs penetration and retention according to the respective biofilm biomass, we quantified the red/green fluorescence intensity for each nanocarrier, with results displayed in Fig. S5. The lack of statistical significance in red/green fluorescence between ssDNA micelles and TDN corroborates their similar penetration and retention capacities, as well as and eliminating the effect of the small biomass difference between the two NPs.

The second notable observation was the strong intensity displayed by TDN-Chit within the biofilm, which was initially surprising given its lower diffusion coefficient. The higher retention is also confirmed by the statistically significant increase in red/green intensity. This phenomenon can be explained by the strong binding affinity of chitosan to bacterial cells. Our study demonstrated the interaction between TDN-Chit and bacteria through a shift in bacterial membrane charge. Consistent with our findings, previous studies have also shown that chitosan binds effectively to microorganisms [88,[107], [108], [109]]. Confocal imaging of the biofilm post-incubation with TDN-Chit showed clusters of bacteria interspersed with empty spaces, a pattern also observed with the SPIM-FCS technique (data not shown). Incubation with the buffer used for these NPs resulted in a smoother and more uniform biofilm mass, suggesting that the presence of chitosan promotes the formation of bacterial clusters. Considering the principle in the use of cation exchanging resins as a method to disrupt the crosslinking in anionic biofilm moieties in the matrix [98], the same theory can be used to explain this observation, where the positive charge of chitosan induces similar changes. Additionally, the observed bacterial charge reversal upon NP incubation with bacteria suggests the adherence of the particles to the bacteria. The sum of these electrostatic interactions likely reduces the mobility of TDN-Chit compared to the other NPs, allowing it to remain within the biofilm matrix even after supernatant removal. This behavior was further supported by the noisy autocorrelation function curve obtained using SPIM-FCS, corroborating the bacterial clumping induced by this NP.

Biofilm maturity can influence matrix composition, and, by extension, influence the interactions between NPs and the matrix, potentially leading to different penetration and retention properties of the structures. For example, different maturation stages can affect biofilm thickness, and it has been hypothesized that thicker biofilms are less dense due to a higher diffusion observed for certain molecules [110,111]. To better understand the influence of different maturity levels on NPs penetration and retention, we incubated less mature biofilms (18h) in the same experimental conditions as for the mature biofilms (72h).

**Fig. 7:**
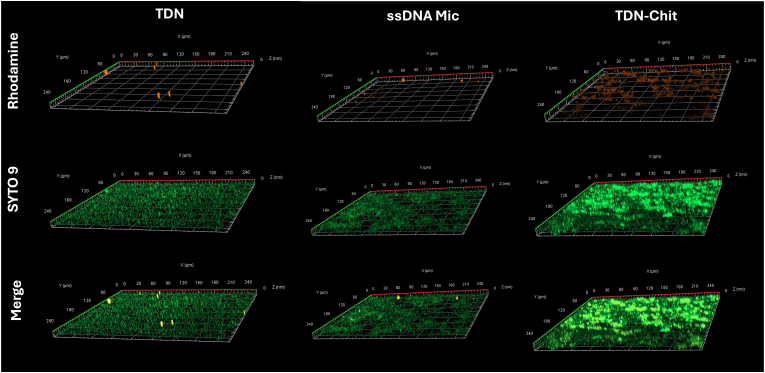
– In vitro penetration evaluation of fluorescently labelled NPs in early-stage (18h) P. aeruginosa biofilms using z-stack images obtained with CLSM. The biofilms were formed for 18h and incubated with the different nanostructures for 2.5h. The bacteria were stained with SYTO 9 (green) and the NPs were rhodamine-labelled (red). Results show similar results as for mature biofilms, where poor penetration of TDN and ssDNA micelles was opposite to the greater penetration ability of TDN-Chit to penetrate early biofilms. (For interpretation of the references to colour in this figure legend, the reader is referred to the Web version of this article.)

As shown in Fig. 7, the behavior of the NPs seems to not be influenced by the degree of biofilm maturation (control images can be found in Fig. S6). We observed a similar trend, where TDN and ssDNA micelles showed low penetration and retention, contrastingly to the strong presence of TDN-Chit, which suggests that penetration might be maturity-independent. A potential explanation for these results is the similar thickness observed between the biofilms at different maturity stages, where the majority of the biofilms were 10–15 μm thick. Interestingly, the effect of chitosan did not appear to have as strong of an impact for early biofilms as compared to mature ones (Fig. S7). Additionally, biofilms pre-treatment both in mature and early stages had similar thickness as post incubation, which were around 10–20 μm, observed upon acquisition of z-stacks with CLSM (Fig. S8). The matrix in *P. aeruginosa* is mainly composed of Pel, Psl, alginate, proteins and eDNA, and the relative contribution of these components to the overall structure is dependent on multiple factors, including maturity stage [112]. We have previously attributed greater penetration of TDN-Chit in comparison to TDN and ssDNA micelles due to greater interactions with negatively charged matrix components. Similar observations have been reported, where chitosan particles enhanced the penetration of an anionic compound due to stronger matrix interactions [113]. Given that the matrix composition changes in different maturity stages, we hypothesize this explains the slight differences of the chitosan coating. Despite this difference, matrix maturity had no visible impact on the penetration and retention ability of the NPs in comparison to biofilms in later development stages.

These findings are intriguing as they demonstrate that DNA-based nanostructures can be tailored for various drug delivery applications in biofilm therapy, exhibiting distinct diffusion, penetration, and retention characteristics within the biofilm structure. This knowledge is highly relevant for future loading of these NPs with antimicrobial compounds, as their behavior can be leveraged to increase efficacy of antibiofilm therapy by altering their physicochemical properties. For example, for antibiotics that have been seen to be sequestered at the biofilm periphery (e.g. tobramycin [5]), encapsulation within ssDNA micelles or TDN would be beneficial due to higher diffusion coefficients, possibly reaching deeper biofilm layers, whereas antibiotics that are challenged by low retention (e.g. fluoroquinolones and macrolides [6,7]) could potentially be loaded into the TDN-Chit as it showed increased retention. Nevertheless, more studies need to be conducted to enhance our understanding on the impact of drug loading in the final NPs.

## 3 Conclusion

This study investigated the diffusion, penetration, and retention behaviors of three distinct DNA-based NPs - ssDNA micelles, TDN, and TDN-Chit - within biofilms. Utilizing a combination of diffusion coefficient determination and confocal laser scanning microscopy (CLSM) for biofilm penetration assays, along with assessments of their interactions with bacterial surfaces, this research marks a significant advancement in understanding the potential of DNA-based NPs as promising agents for future drug delivery systems in bacterial biofilms.

Our findings revealed that while ssDNA micelles and TDN exhibited similar diffusion coefficients and degrees of penetration within the biofilms, TDN-Chit demonstrated a significantly reduced diffusion coefficient (attributed to larger size and positive charge), however displayed greater retention. The ability of the NPs to penetrate biofilms was found to be independent of the maturity stages, with similar tendencies found in both early and mature stages. Furthermore, the study highlighted that the shape of the NPs did not significantly influence their diffusion or penetration profiles, suggesting that size and surface charge are still more critical factors in determining the interactions of DNA-based NPs with biofilm structures.

Overall, this study provides crucial insights into the field of NP-biofilm interactions, particularly laying the ground for the rational design of DNA-based NP drug delivery systems in the context of biofilm therapy. The observed differences in the behavior of these NPs are particularly significant as they directly influence the selection of drug to be encapsulated within these structures. Each NPs unique interaction with the biofilm matrix suggests that the efficacy of the loaded therapeutic agent could vary, necessitating a tailored approach to drug delivery. However, further research is essential to fully understand these interactions and optimize the therapeutic potential of DNA-based NPs in treating biofilm-associated infections. Additionally, it is important to note that direct extrapolation of our findings to other biofilm-forming bacterial species should be approached with caution, as variations in the structural and compositional characteristics of biofilms among different pathogens as well as the in mixed species biofilms may result in different observations.

## 4 Experimental

### 4.1 Materials

Synthetic oligonucleotides (see Table S1 for sequences and modifications) were ordered from Integrated DNA Technologies, Inc (CA, USA). The strain of *Pseudomonas aeruginosa* (PAO1, ATCC 10145-GFP) was obtained from American Type Culture Collection (DC, USA). Cation-adjusted Mueller Hinton Broth (MBH), Sodium acetate trihydrate, Tris, NaCl and MgCl_2_ were purchased from Sigma-Aldrich (MO, USA). Luria broth (LB) medium was purchased from Invitrogen. Acetic acid glacial was acquired from VWR International (Fontenay-sous-Bois, France). Chitopharm™ M − Chitosan with medium molecular weight (200–350 kDa) and a deacetylation degree of >80 % was generously provided by Chitinor (Tromsø, Norway).

### 4.2 Nanoparticle fabrication

The preparation of different nanostructures was based on previously published methods with some modifications. The fabrication of DNA Tetrahedron (TDN) was prepared similarly to the work published by Sun et al. [114], where each strand was added to a final concentration of 1 μM in TM buffer (10 mM Tris and 50 mM MgCl_2_), heated to 95 °C for 5 min and allowed to cool down to 4 °C for 30 min, and then diluted to 0.5 μM in 1xTM. For the micelle preparation (ssDNA micelles), a previously used method was employed [36], in which the structures spontaneously self-assemble when the cholesterol conjugated single-stranded DNA is added to 1x micelle buffer (MB) (2.5 mM NaCl, 1.25 mM MgCl_2_, 0.05 M sodium acetate, pH 4.5) to a final concentration of 20 μM. For the chitosan coating of the TDN, nanostructure, a chitosan solution was initially prepared by making a 1.6 % stock solution of medium weight chitosan in 1 % (v/v) acetic acid in MilliQ water. The solution was further diluted with 1x TM to a final concentration of 0.1 %, which was filtered through a 0.22 μm filter immediately before use. To coat the TDN, equal volumes of TDN at 1 μM and the chitosan (0.1 % w/v) were added either manually or with the microfluidic device Fluidic 187 (ChipShop, Germany). When using this device to mix the two components, the TDN and chitosan were added with two different syringes, each containing one of the components, that were attached to a Pump (33DDS, Harvard Apparatus, US) with control over speed of addition.

For clarity purposes, the TDN-Chit translates to a final TDN concentration of 0.5 μM and 0.05 % of chitosan. All particles were used at these concentrations in all experiments.

### 4.3 Nanostructures' characterization

The nanostructures were characterized in terms of hydrodynamic diameter (size), polydispersity index (PDI) and zeta potential using dynamic light scattering (DLS) with the Zetasizer Nano Zen 2600 (Malvern Instruments Ltd, Malvern, UK). The measurements were performed at 25 °C and presented as mean ± standard deviation (SD).

### 4.4 Critical micelle concentration (CMC) determination

The determination of critical micelle concentration was conducted with UV–visible spectrophotometry according to previously established methods [44,45], where the micellization process induces changes in the linearity of absorbance. In brief, different concentrations of the cholesterol-modified ssDNA sequences were sequentially diluted in 1x MB and the absorbance was measured at 280 nm using a microplate reader (Spark, Tecan).

### 4.5 Nanoparticles’ interaction with bacterial membrane

The bacterial strain used in this study was *P. aeruginosa* GFP ATCC 10145-GFP. Aliquots were streaked on Luria broth (LB) agar plates and incubated at 37 °C overnight. Afterwards, one bacterial colony was used to inoculate 5 mL of sterile LB media with 200 μg/mL of carbenicillin and incubated at 37 °C at 100–150 rpm overnight. The bacteria were spined down at 3000 rpm for 5 min, and the supernatant was discarded. The bacteria were then resuspended in 1x PBS and the optical density (OD) was adjusted to 0.1 at 600 nm. Next, 100 μL of each NPs suspension or respective buffer (as control) were added to 100 μL of the bacterial suspension and the zeta potential was recorded using dynamic light scattering (DLS) with the Zetasizer Nano Zen 2600 (Malvern Instruments Ltd, Malvern, UK).

### 4.6 SPIM-FCS

For biofilm formation, a single colony of *P. aeruginosa* PAO1 was added to 5 mL of Luria Broth (LB) medium and incubated overnight at 220 rpm at 37 °C, further diluted into an optical density (OD) of 0.3 before being added to the wells. The biofilms were grown in accordance to similar studies [89] with some modifications. FEP films were attached to the sample chamber, treated with 200 μL of a solution of 0.01 % Poly-l-Lysine for 5 min and left to dry at room temperature for 2 h. Afterwards, 400 μL of the bacterial suspension was added to each FEP well and grown for 72h at 30 °C. The biofilms were washed once with 1x PBS and 200 μL of the NPs suspension was added to each well, where the final concentration of NPs was the same as for previous studies, but each contained a rhodamine-labelled strand in a final concentration of 0.1 μM to ensure the same signal from each particle. The tetrahedral DNA nanostructure (TDN) was constructed using four single DNA strands: T1, T2, T3, and T4 (refer to Supplementary Information Table 1). For fluorescent labeling, only the T1 strand was modified with a fluorophore (T1-Fluorophore). The assembly was initiated by combining T1-Fluorophore at a concentration of 0.2 μM with unlabeled T1 at 0.8 μM, achieving a total T1 concentration of 1 μM. The remaining single-stranded DNA (ssDNA) components, T2, T3, and T4, were each added to a final concentration of 1 μM to ensure uniformity across the assembly. Following assembly, the TDN structure was either diluted 1:1 in 1xTM buffer (resulting in TDN) or coated with chitosan as previously described (resulting in TDN-Chit).

For the micelle assembly, a similar strategy was employed. The structure was formed by incorporating 0.1 μM of rhodamine cholesterol-modified ssDNA alongside 19.9 μM of standard cholesterol-modified ssDNA, resulting in a final concentration of 20 μM.

The biofilms were imaged after 0–5 h of incubation, which depended on the required time to acquire the data. Solution measurements were conducted separately in untreated sample chambers.

A laser power of 30 mW was used for acquisition during the measurements. The illumination objective was LSFMx10/NA0.2 and a WPlan-Apochromat x63/NA1.0 was used as detection objective. The detection of fluorescence was filtered at 500–530 nm for GFP and 580–627 nm for Rhodamine. A frame length of 50 000 with 0.8 ms exposure time was used, and analysis of the data was conducted with ImageJ plugin Imaging FCS 1613. Polynomial order 4 bleach correction was used to correct for bleaching of fluorophores during acquisition. A 4x4 in-camera pixel binning was used in order to obtain a better signal. The fitting model for calculation of diffusion coefficient can be found in the imFCS plugin manual.

### 4.7 Determination of nanostructure penetration via confocal microscopy

For biofilm formation, a single colony of *P. aeruginosa* PAO1 was inoculated in 5 mL of Luria Broth (LB) medium at 37 °C and 150 rpm overnight. The suspension was adjusted to an optical density (OD) of 0.3 at 600 nm, and 400 μL of this suspension were added to each well of a CC^2^™treated 8-well glass slide. The biofilms were grown at 30 °C for 72h. The supernatant was then removed, and the biofilms were washed with 1x PBS to remove planktonic bacteria. Afterwards, NP suspension was added to the wells (200 μL), in the same final NP concentration as in the previous experiments, where each had a final rhodamine-labelled strand of 1 μM to ensure consistent intensity signals between NPs. In this experiment, the same logic as for the SPIM-FCS technique was used for the fabrication of fluorescently labelled NPs. In brief, 1 μM of T1-Fluorophore was added to equimolar concentrations of T2, T3 and T4. After assembly, the structure was either diluted in 1xTM buffer (TDN) or chitosan in the same concentrations as for other experiments (TDN-Chit). For micelle formation, 1 μM of rhodamine cholesterol-modified ssDNA was added simultaneously with 19 μM of standard cholesterol-modified ssDNA to a buffer solution, resulting in a final micelle concentration of 20 μM.

The NPs or their respective buffers (as control) were incubated for 2.5 h at 30 °C protected from light. Then, the supernatant was removed, and the biofilms were stained with aliquots of SYTO9 according to the manufacturer's instructions and incubated for 20 min at room temperature, protected from light. The biofilms were gently washed again with 1xPBS to remove excess dye and covered with a glass coverslip. The penetration of the NPs was then investigated with Confocal Laser Scanning Microscopy (CLSM) with the Zeiss LSM 800 (Carl Zeiss, Oberkochen, Germany) using a water 25x objective lens. For the quantification of red/green intensity, ImageJ was used to analyze fluorescence intensity of at least three z-stacks per nanoparticle.

### 4.8 Statistical analyses

The statistical analyses were conducted using Graphpad Prism (Version 10, GraphPad Software Inc., CA, USA). The student's *t*-test was employed between two groups with statistical difference. One-way Anova or two-way Anova with Turkey's *post hoc* analysis was performed for data consisting of three or more groups. Results are presented as mean ± standard deviation and *p* values of <0.05 were considered significant.

## CRediT authorship contribution statement

**Alexandra Sousa:** Writing – review & editing, Writing – original draft, Methodology, Investigation, Formal analysis, Data curation, Conceptualization. **Rutuparna Kulkarni:** Writing – review & editing, Writing – original draft, Methodology, Investigation, Formal analysis. **Mona Johannessen:** Writing – review & editing. **Thorsten Wohland:** Writing – review & editing, Resources, Methodology, Investigation, Conceptualization. **Nataša Škalko-Basnet:** Writing – review & editing, Supervision, Project administration. **Sybil Obuobi:** Writing – review & editing, Supervision, Project administration, Funding acquisition, Conceptualization.

## Declaration of generative AI

During the preparation of this work, the authors used ChatUiT to improve readability. After using this tool, the authors reviewed and edited the content as needed and take full responsibility for the content of the published article.

## Declaration of competing interest

The authors declare that they have no known competing financial interests or personal relationships that could have appeared to influence the work reported in this paper.

## Data Availability

Data will be made available on request.
