# Supplementary material for: Decoding interactions between biofilms and DNA nanoparticles
Source: Biofilm. 2025 Feb 6;9:100260. doi: 10.1016/j.bioflm.2025.100260 (PMC11871490; doi:10.1016/j.bioflm.2025.100260)
Supplement: Multimedia component 1 [file mmc1.docx]

**Supporting Information**

**Decoding interactions between biofilms and DNA nanoparticles**

**Results:**

**Table S1 -** Sequences of ssDNA used in the synthesis of different DNA-based nanoparticles

| ***Nanoparticle*** | ***Component*** | ***Sequence*** |
| --- | --- | --- |
| **Micelle** | **Chol_ssDNA** | 5´- ATCGGTAGGGTGTCA/3CholTEG/-3´ |
|  | **Chol_ssDNA-Fluorophore** | 5'-RhoR-XN-ATCGGTAGGGTGTCA-3CholTEG-3´ |
| **Tetrahedron** | **T1** | 5´-A GTC TTC GTC CTT ATC GGT AGA GGT GCT GAG CGG AAT CCT GA A AGT GTA CAA GGT ATC TCG AC-3´ |
|  | **T2** | 5´-A CTA CCG ATA AGG ACG AAG AC A CTA AGA CTG GAG GAC CGA TG A CGA GCA GAC AAC AGC GC-3´ |
|  | **T3** | 5´-A TCA GGA TTC CGC TCA GCA CC A GCG CTG TTG TCT GCT CG A ATG TAC CAT CGT TAC TAG AT-3´ |
|  | **T4** | 5´-A CAT CGG TCC AGT CTT AG A GTC GAG ATA CCT TGT ACA CT A ATC TAG TAA CGA TGG TAC AT-3´ |
|  | **T1-Fluorophore** | 5´-/5RhoR-XN/A GTC TTC GTC CTT ATC GGT AGA GGT GCT GAG CGG AAT CCT GAA AGT GTA CAA GGT ATC TCG AC -3’ |

**Figure S1** – CMC determination for ssDNA micelles

**
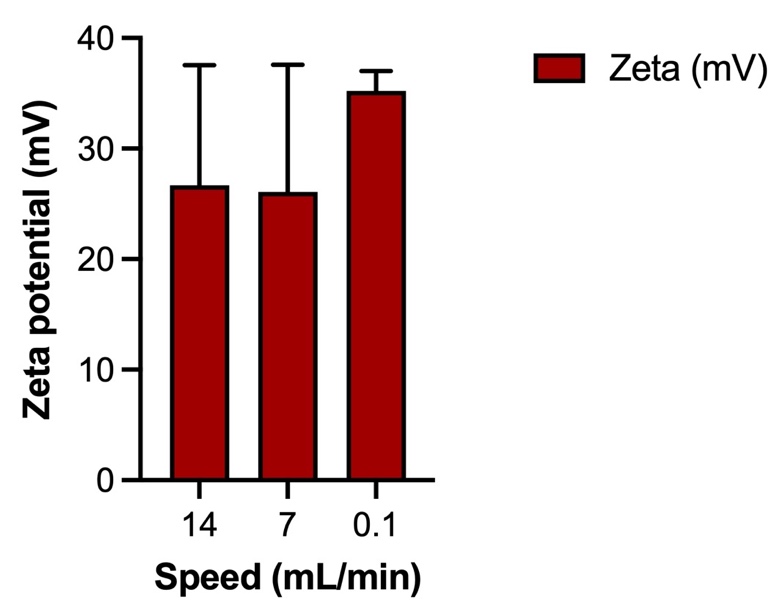
**

**Figure S2 –** Zeta potential of TDN-Chit synthesized using different speed with the microfluidics device.


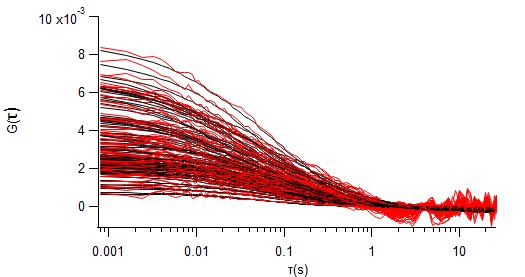


**Figure S3** – Autocorrelation curve of TDN-Chit using two-particle fitting.


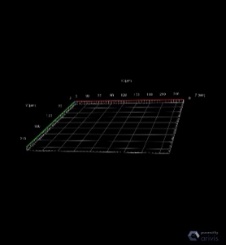

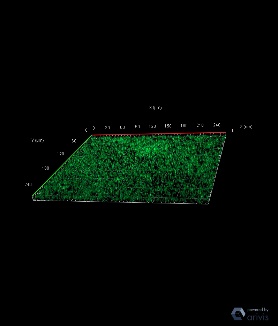


Green

Red

Merge

**Control for ssDNA micelles**

**Control for**

**TDN**

**Control for**

**TDN-Chit**


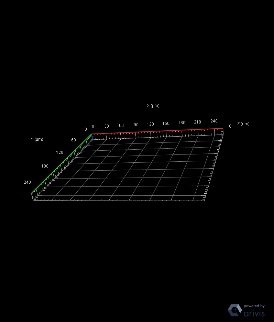

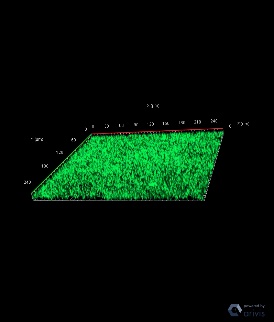

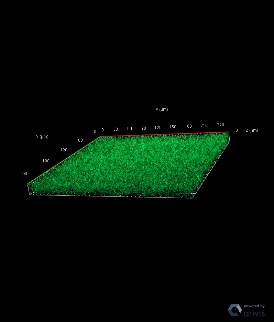

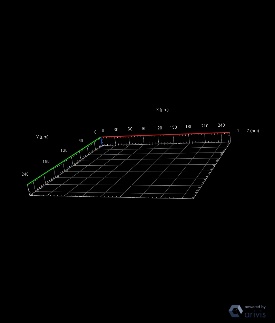

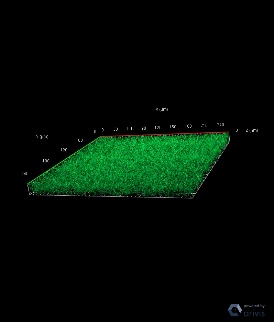

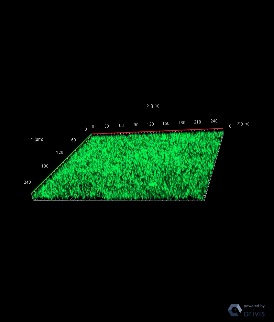

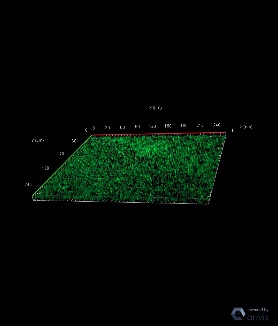


**Figure S4** – CLSM images of *P. aeruginosa* mature (72h) biofilms incubated with respective buffers of each nanoparticle (controls).

**Figure S5** – Nanoparticle penetration and retention analysis through the quantification of red (nanoparticle) to green (biofilm) fluorescence intensity and respective proportion using ImageJ.


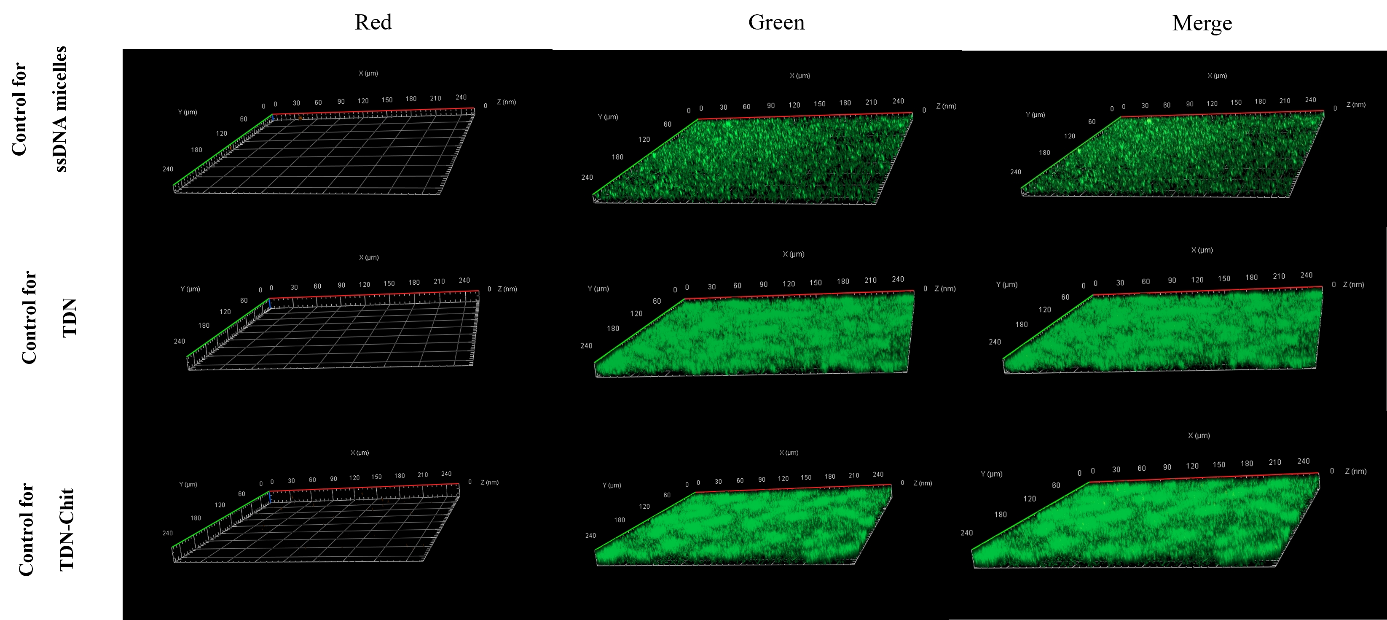


**Figure S6** – CLSM images of early-stage (18h) *P. aeruginosa* biofilms incubated with respective buffers of each nanoparticle (controls).


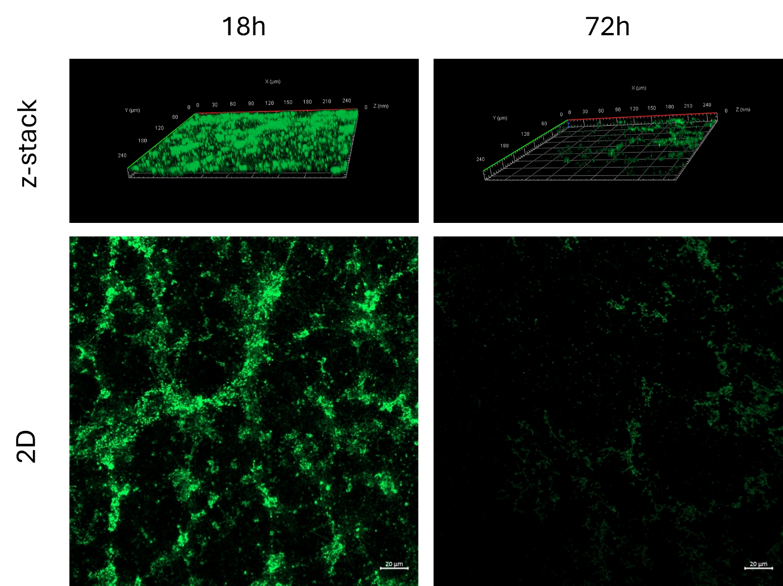


**Figure S7** – CLSM z-stack (top) and 2D (bottom) images of early (left) and mature (right) stages of *P. aeruginosa* biofilms incubated with chitosan only at the same concentration as used for the TDN coating.


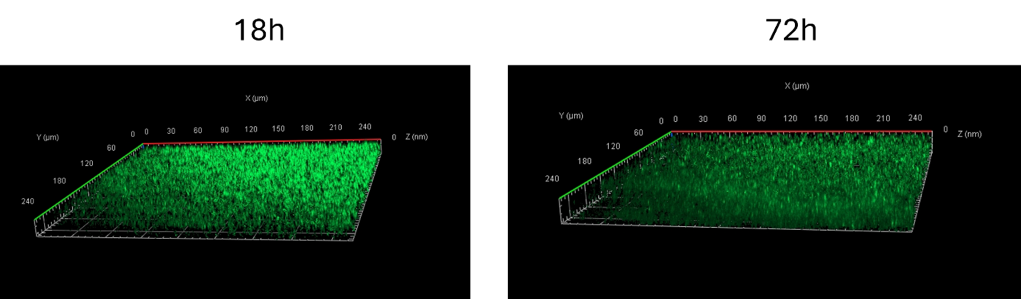


**Figure S8** – CLSM images of early (left) and mature (right) stages of *P. aeruginosa* biofilms pre-incubation with NPs.
